# Vaginoscopic Incision of Vaginal Septum With Preservation of the Hymen in a Child With Obstructed Hemi-Vagina Ipsilateral Renal Agenesis (OHVIRA) Syndrome

**DOI:** 10.7759/cureus.30450

**Published:** 2022-10-19

**Authors:** Wael M Moneir, Fayez Almodhen, Jamila Almaary, Zahra Almatar, Abdullah Alaqeel

**Affiliations:** 1 Pediatric Urology, King Abdulaziz Medical City Riyadh, Riyadh, SAU; 2 Pediatric Urology, King Abdullah Specialized Children Hospital, King Abdulaziz Medical City, Ministry of National Guard Health Affairs, Riyadh, SAU; 3 Pediatric Surgery, King Abdulaziz Medical City Riyadh, Riyadh, SAU; 4 Pediatric Surgery, King Faisal Specialist Hospital and Research Centre, Riyadh, SAU

**Keywords:** minimally invasive, vaginoscopy, vaginal septum, mullerian anomalies, ohvira

## Abstract

Obstructed hemi-vagina ipsilateral renal agenesis (OHVIRA) syndrome, also known as Herlyn-Werner-Wunderlich syndrome (HWWS) is a rare variant of Mullerian duct anomalies that usually presents after menarche. Although there is increasing awareness about OHVIRA syndrome, high suspicion is needed for the diagnosis. Awareness of the syndrome is crucial for the management and to prevent serious complications.

Surgical techniques and age at the surgery are still debatable but, minimally invasive vaginoscopic resection of the vaginal septum should be considered when feasible as it not only allows division of septum with preservation of hymen, but it provides excellent visualization, is less traumatic, and has promising postoperative outcomes. Hymenal integrity is of great concern in specific populations with cultural values.

In this report, we present the case of a 24-month-old girl diagnosed with OHVIRA syndrome during a routine follow-up for renal agenesis and was managed with vaginoscopic incision of the vaginal septum using a pediatric cystoscope while maintaining the hymenal integrity.

## Introduction

Purslow described the Herlyn-Werner-Wunderlich syndrome (HWWS) in 1922 [1]. The syndrome was later named obstructed hemi-vagina ipsilateral renal agenesis (OHVIRA) syndrome as suggested by Smith and Laufer in 2007. It is a rare congenital anomaly that is characterized by eccentric development of both the Mullerian and Wolffian ducts. OHVIRA is an acronym that stands for obstructed hemi-vagina and ipsilateral renal agenesis [2]. The syndrome is usually discovered after menarche in adolescent females and its symptoms are infrequently encountered in prepubertal girls [3,4]. Diagnosis of OHVIRA in prepubertal girls needs a high level of suspicion to allow prompt recognition and timely management to prevent complications and preserve reproductive capacity [5]. Magnetic resonance imaging (MRI) and ultrasound remain the main modalities for investigation, while laparoscopy is sometimes needed to confirm the diagnosis [6]. While vaginoplasty with resection of the vaginal septum is usually the management of OHVIRA syndrome [2], Minimally invasive procedures performing resection or complete incision of the vaginal septum are currently advocated for the treatment of the obstructed hemi-vagina [7].

In this report, we present a case of OHVIRA syndrome diagnosed in a two-year-old girl, who was managed with a vaginoscopic incision of the vaginal septum while preserving the hymen for sociocultural concerns.

This article is accepted for presentation as a meeting abstract at the 7th World Congress of the World Federation of Associations of Pediatric Surgeons (WOFAPS), which will take place in Prague, Czech Republic, in October 2022.

## Case presentation

**Figure 1:**
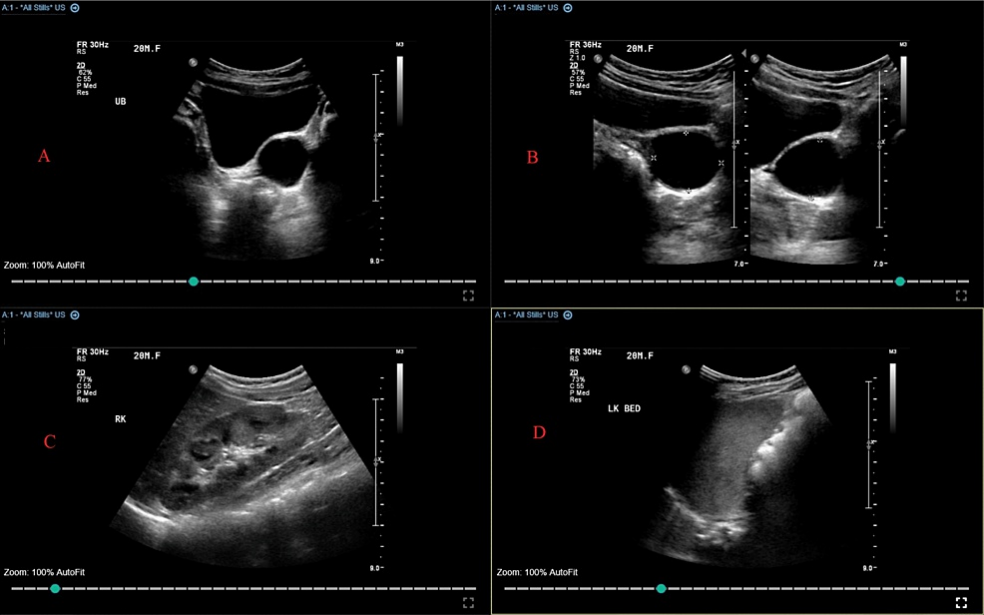
Renal ultrasonography scan showing pelvic cystic lesion posterior to the bladder (A, B), normal right kidney (C), and absent left kidney (D)

A 24-month-old girl who has been under regular follow-up with pediatric urology for an antenatally diagnosed left renal agenesis was discovered incidentally to have a pelvic cystic lesion posterior to the bladder measuring 2.6x1.9 cm (Figures 1A-1D).

**Figure 2:**
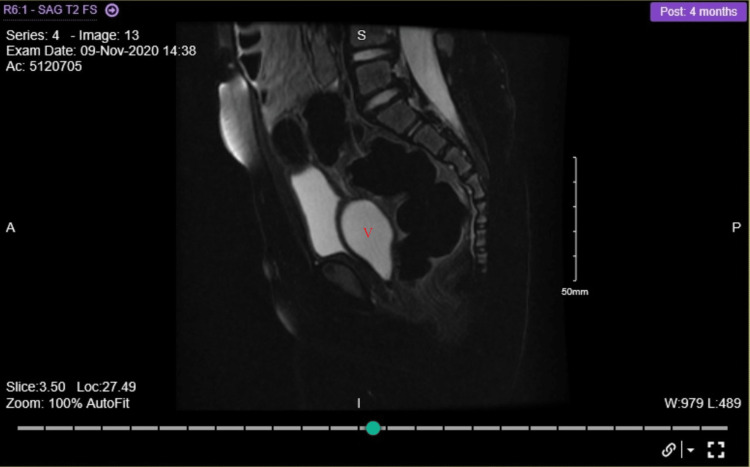
Magnetic resonance imaging of the pelvis demonstrating a fluid-filled vagina (red V)

The cyst was discovered during her routine renal ultrasonography (RUS) scan. Her previous RUS scans were normal apart from a spontaneously resolved Society for Fetal Urology (SFU) grade II hydronephrosis (APRPD 4 mm) of her solitary right kidney at the age of four months. Her voiding cystourethrogram was negative for vesicoureteral reflux, and her renal DMSA scan showed a normal functioning right kidney. The patient was completely asymptomatic, and never had a urinary tract infection or urinary symptoms. A repeated US showed progressive enlargement of the pelvic cyst (2.2x3.6 cm). MRI scan revealed a fluid-filled vagina (hydrocolpos) with suspicion of a vaginal septum (Figure 2).

**Figure 3:**
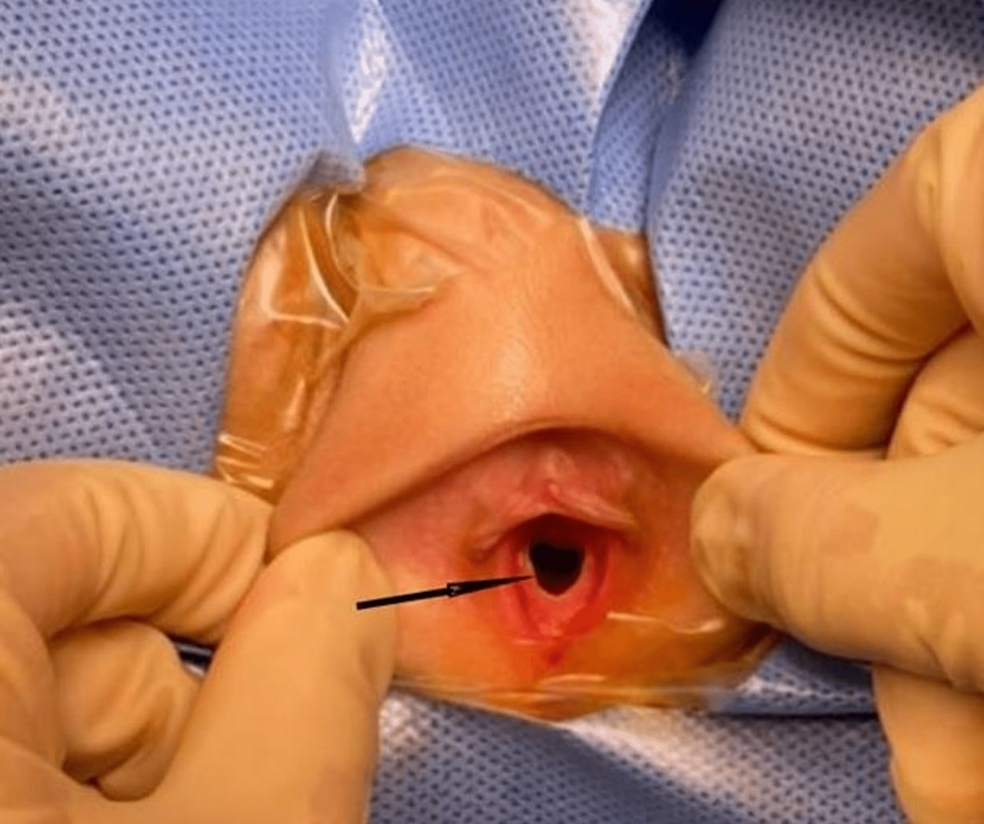
Pelvic examination under anesthesia showing normal female external genitalia and an intact hymen

Findings were suspicious for OHVIRA syndrome, the family was counseled regarding the possible diagnosis. The natural history of the disease, spontaneous resolution rates of hydrocolpos, possible complications, and potential lines of management including conservative follow-up with future vaginoplasty at puberty were explained in detail to the family. The patient’s family demanded definitive surgical management without delay but, they had a concern regarding hymen integrity to socio-cultural beliefs. After obtaining informed consent, the patient underwent an examination under anesthesia with diagnostic cystoscopy and vaginoscopy. After induction of anesthesia, her examination showed normal female external genitalia with intact, annular hymen (Figure 3).

**Figure 4:**
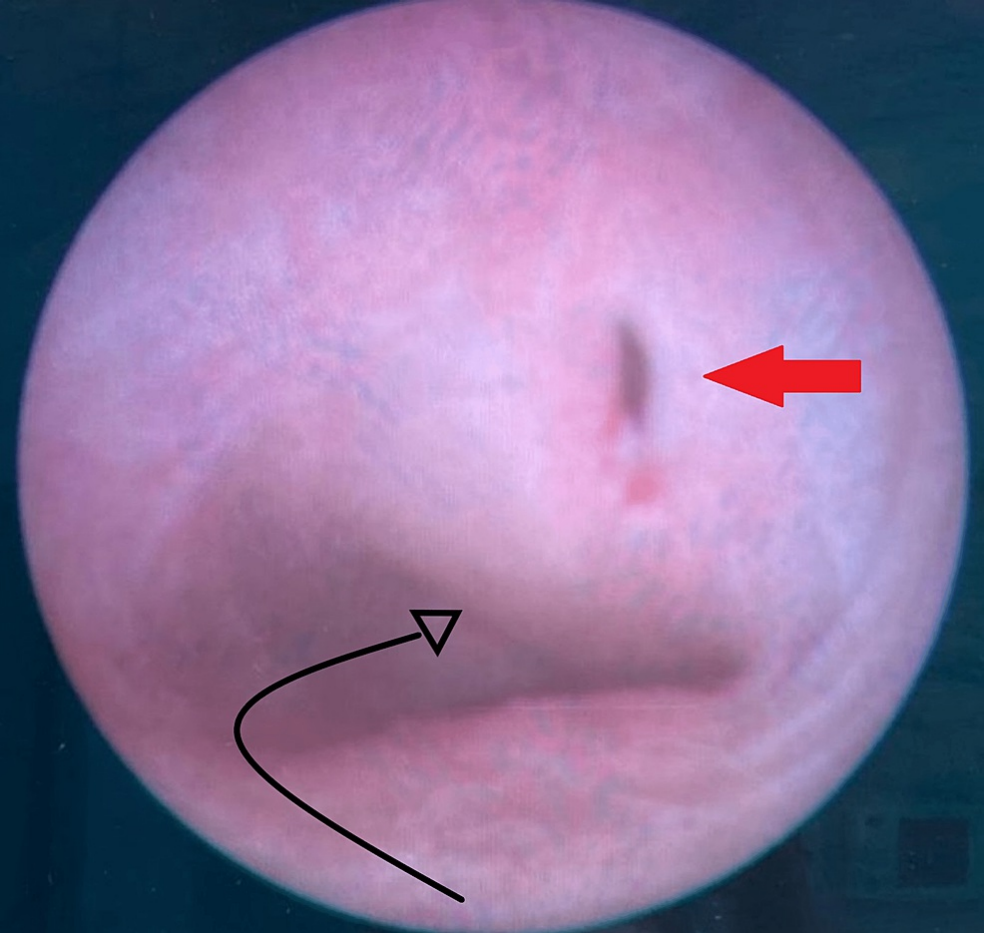
Vaginoscopic view of the bulging vaginal septum with puncture site (red arrow), curved black arrow indicates the location of the right cervix behind the septal bulge

Cystoscopy revealed a normal right ureteric orifice and a normal bladder, bladder neck, and urethra. No Left ureteric orifice was identified. The bladder was then filled with normal saline to improve visualization by transabdominal ultrasound during the procedure. A diagnostic 8.5Fr pediatric cystoscope (Karl-Storz Company, Germany) was inserted into the vagina carefully through the hymenal ring. An assistant used a gauze pad around the cystoscope and applied pressure toward the orifice of the vagina to reduce the leakage of the irrigation fluid while the operator manipulated the cystoscope. The vaginal septum was easily identified as bulging from the left lateral wall of the vagina with a cervix located posterior and to the right of the bulge. Under ultrasound guidance, the vaginal septum was incised using a 9Fr resectoscope with a miniature straight, forward telescope and heat-cutting loop (Karl-Storz Company, Germany), and the obstructed hemivagina was drained (Figure 4).

**Figure 5:**
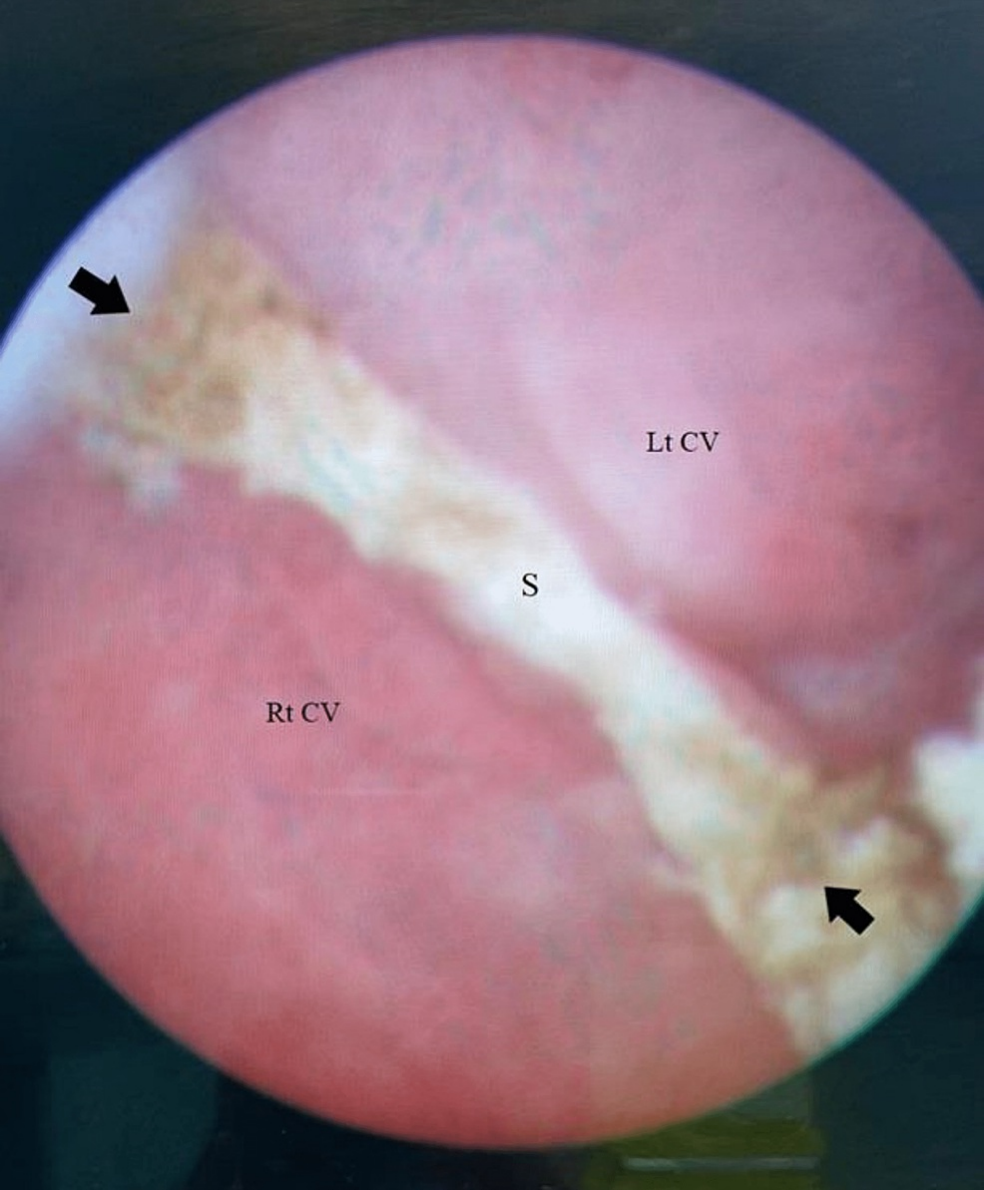
Vaginoscopic view after complete septal resection (S) and demonstrating the right cervix (Rt CV) and the left cervix (Lt CV) The view was taken from a video clip and is thus of poor quality

8Fr rigid pediatric cystoscope was inserted through the septotomy revealing another cervix to the left of the midline. The incision was extended cephalically to approximately 3mm below the cervices and caudally to the junction of the septum and the lateral vaginal wall resulting in an incision of the entire septum (Figure 5).

**Figure 6:**
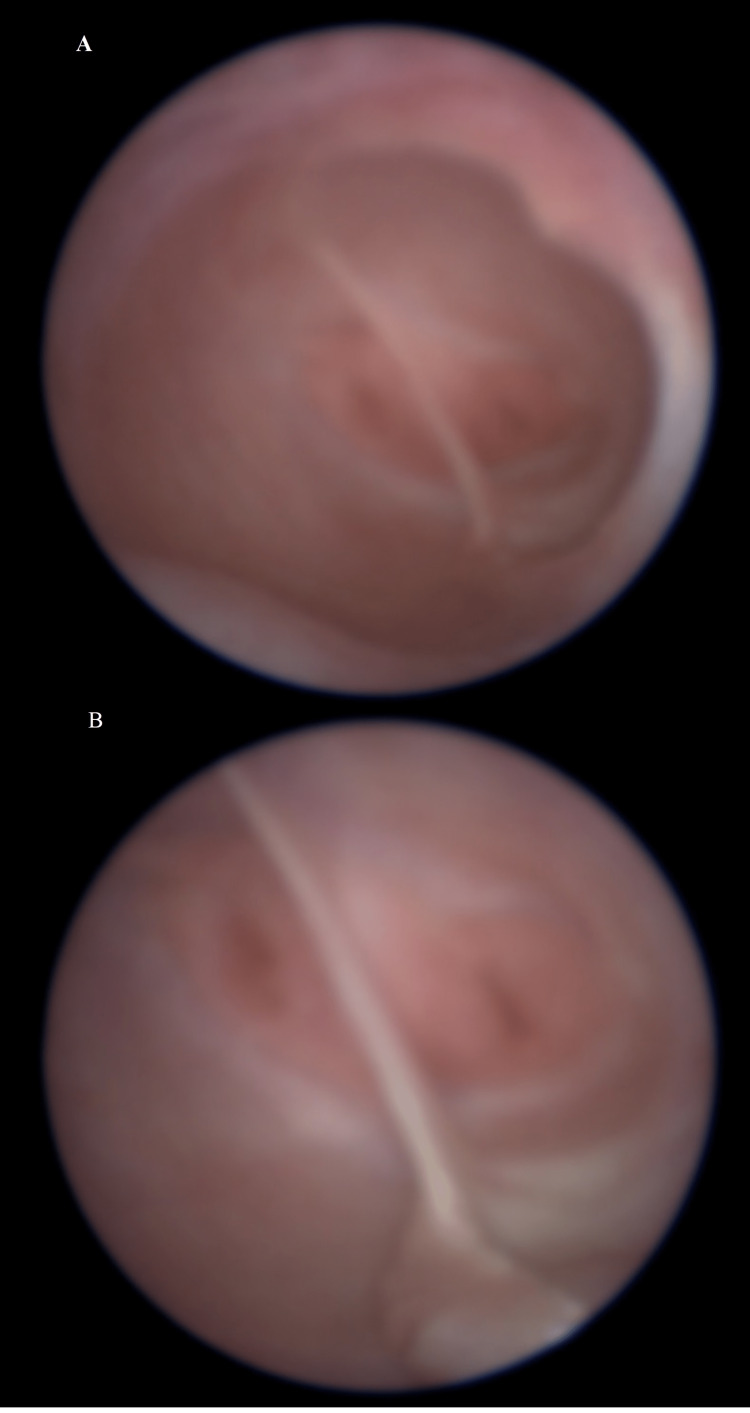
(A, B) Follow-up vaginoscopic view, six weeks after septal resection, showing a single vagina with two cervices The view was taken from a video clip and is thus of poor quality

The right cervix previously hidden by the vaginal septum was easily seen. Hemostasis was maintained when needed. No catheter, packing, or drain was used. The final vaginoscopic appearance showed a single vaginal vault, two cervices, and an intact hymenal ring. No ectopic ureteric orifice was seen. The patient recovered well postoperatively. She was discharged two days after the procedure; a second look vaginoscopy was scheduled three months later. At the follow-up vaginoscopic examination, no residual septum or stenotic ring was noticed, wide vagina with two cervices and an intact hymenal ring were visualized (Figures 6A, 6B).

The patient was discharged on the same day. The patient will have a regular follow-up with periodic pelvic and renal ultrasonography scans, and she is planning for a follow-up vaginoscopy and referral to gynecology at puberty.

## Discussion

Mullerian tract abnormalities have been reported in 2% to 3% of women [8], of these anomalies, OHVIRA is a rare category that involves only 5% of the affected individuals [9]. OHVIRA syndrome is characterized by abnormal development of both the Mullerian (paramesonephric) and Wolffian (mesonephric) ducts [2]. The pathogenesis of the syndrome is not clearly understood but is considered multifactorial. The mesonephric ducts will give rise to the kidney and ureter, it also induces the normal development of paramesonephric ducts. Hence, abnormal development of the Wolffian ducts leads to unilateral renal agenesis and imperforated hemi-vagina associated with OHVIRA syndrome [10].

Smith and Laufer proposed the acronym OHVIRA to describe the distinctive triad of this syndrome including obstructed hemi-vagina and ipsilateral renal agenesis [2]. Recently, with the increasing number of cases that do not fit the classic definition of OHVIRA syndrome, it was suggested to incorporate a spectrum of ipsilateral renal anomalies in the definition of the syndrome including ipsilateral dysplastic, atrophic kidney and ectopic ureter [8,11,12]. On the other hand, septated uterus and single uterus have been rarely reported with the syndrome [13,14].

Classically, patients with OHVIRA present in their adolescence with progressive cyclic pelvic pain, dysmenorrhea, or pelvic mass a few months after menarche [2]. In the non-classic presentation, as with our patient, patients may initially be discovered during neonatal follow-up for prenatally diagnosed single or dysplastic kidneys [12], or with the presence of vulvar mass over the vaginal introitus [12,15]. Prompt recognition of OHVIRA syndrome and time management is required to prevent complications such as endometriosis and its adverse impacts on future fertility [5,16,17].

Ultrasonography and MRI are usually the tools to investigate pelvic pathology and Mullerian duct anomalies, pelvic US is frequently the first step due to its availability and non-invasiveness. Unfortunately, ultrasonography is operator-dependent, and diagnosing OHVIRA syndrome can be tricky. For such reasons, MRI remains the cornerstone imaging study for the diagnosis of the syndrome. This is due to its ability to accurately depict pelvic organs, detect associated anomalies, and its multiplanar capacity. MRI is usually essential for the preoperative planning of management [6,16,17]. Laparoscopy plays a role in the diagnosis of OHVIRA in the case of the doubt after MRI or when MRI is not available [2,16].

Management of the “O” component of the syndrome requires the drainage of the accumulated fluids and removal of the vaginal septum. Historically, single-stage or two-staged vaginoplasty is considered to be the definitive management [2]. However, vaginoplasty can be traumatic when exposure to the underdeveloped vagina is needed for the surgical treatment of adolescents or children affected [7]. In such cases, minimally invasive vaginoscopic resection of the vaginal septum is currently advocated, and short-term results are promising [7,18].

Guidance regarding the management and timing of intervention for asymptomatic OHVIRA in the pediatric age group is lacking. A recent report suggested that most children with asymptomatic OHVIRA can be safely managed conservatively without the need for vaginal surgery, and pre-symptomatic elective surgery can be considered at puberty to prevent complications that cause fertility and renal impairment [19]. In our case, despite the increasing hydrocolpos, the patient remained asymptomatic. The family’s decision played an important role in deciding on surgery.

The hymenal tissues are sensitive to forceful manipulation and wide movements needed during the resection of the vaginal septum in the traditional vaginoplasty, and as virginity does matter for certain populations, minimally invasive procedures using flexible or small rigid scopes should be considered especially when cultural values of maintained hymenal integrity are very important for the patients and their families [7,20].

Since vaginoscopic intervention for OHVIRA had been recently introduced, to the best of our knowledge, there is no data regarding long-term outcomes for prepubertal patients managed with this modality. Thus, long-term follow-up is warranted. Periodic clinical reassessment, renal US, pelvic US, and vaginoscopy with a gynecologist at puberty are our suggested plans of follow-up.

## Conclusions

Although there is increasing awareness about OHVIRA syndrome, high suspicion is still needed for the diagnosis due to atypical presentations of the disease. Vaginoplasty, the standard management of the obstructing vaginal septum can be traumatic, especially in young patients with an immature vagina. Minimally invasive vaginoscopic resection of the vaginal septum should be considered when feasible as it does not only allow division of the septum with preservation of the hymen, but it provides excellent visualization, is less traumatic, and has promising postoperative outcomes. However, prepubertal girls managed endoscopically should be followed to assess the long-term outcomes. Timing of intervention can be challenging, especially in asymptomatic young patients. Parental preferences might affect decision-making. Hymenal integrity is of great concern in specific populations with cultural values that should be respected.
